# Use of Machine Learning and Wearable Sensors to Predict Energetics and Kinematics of Cutting Maneuvers

**DOI:** 10.3390/s19143094

**Published:** 2019-07-12

**Authors:** Matteo Zago, Chiarella Sforza, Claudia Dolci, Marco Tarabini, Manuela Galli

**Affiliations:** 1 Dipartimento di Elettronica, Informazione e Bioingegneria, Politecnico di Milano, 20133 Milano, Italy; 2 Fondazione Istituto Farmacologico Filippo Serpero, 20159 Milano, Italy; 3 E4Sport Lab, Politecnico di Milano, 20133 Milano, Italy; 4 Dipartimento di Scienze Biomediche per la Salute, Università degli Studi di Milano, 20133 Milano, Italy; 5 Dipartimento di Meccanica, Politecnico di Milano, 20129 Milano, Italy

**Keywords:** supervised learning, changes of direction, IMU, mechanical work

## Abstract

Changes of directions and cutting maneuvers, including 180-degree turns, are common locomotor actions in team sports, implying high mechanical load. While the mechanics and neurophysiology of turns have been extensively studied in laboratory conditions, modern inertial measurement units allow us to monitor athletes directly on the field. In this study, we applied four supervised machine learning techniques (linear regression, support vector regression/machine, boosted decision trees and artificial neural networks) to predict turn direction, speed (before/after turn) and the related positive/negative mechanical work. Reference values were computed using an optical motion capture system. We collected data from 13 elite female soccer players performing a shuttle run test, wearing a six-axes inertial sensor at the pelvis level. A set of 18 features (predictors) were obtained from accelerometers, gyroscopes and barometer readings. Turn direction classification returned good results (accuracy > 98.4%) with all methods. Support vector regression and neural networks obtained the best performance in the estimation of positive/negative mechanical work (coefficient of determination R^2^ = 0.42–0.43, mean absolute error = 1.14–1.41 J) and running speed before/after the turns (R^2^ = 0.66–0.69, mean absolute error = 0.15–018 m/s). Although models can be extended to different angles, we showed that meaningful information on turn kinematics and energetics can be obtained from inertial units with a data-driven approach.

## 1. Introduction

Changes of direction (CoD) and cutting maneuvers are basic locomotor actions in team sports, implying high physiological and mechanical load [1,2,3]. High-intensity and abrupt sidestepping is the most frequent cause for non-contact ligamentous injuries at the knee level, involving primarily anterior cruciate ligament lesions, and secondarily meniscal or medial collateral ligament strains [4]. The amount of deceleration required in sidestep cutting is related to the angle and speed of approach and has been associated to the likelihood of knee injuries [5,6]. CoDs also have a high associated metabolic cost, impacting on the energetic requirements of exercise [7,8].

The mechanics and neurophysiology of CoDs have been accurately described primarily in laboratory conditions, unveiling joint kinematics and loads as a function of the running angle and technique [1,9,10,11], foot-landing strategies [12], muscular activations [3,13], and response to fatigue [14,15]. In addition, we recently proposed an algorithm to estimate the energy cost of running with repeated 180 degree-CoDs [16]: the external mechanical energy associated to the trajectory of a body’s center of mass was combined with the knee flexion angle and ground contacts to provide an estimation of the positive (concentric) and negative (eccentric) muscular work.

Although promising, this method was limited by the obtainment of full-body kinematics with optical motion capture systems, being therefore confined to laboratory conditions. Rather, gathering reliable information on the side, speed and energetics of 180-degree sidestep CoDs in realistic on-the-field conditions would better help in monitoring the energetic, physiological and mechanical load, as well as to prevent overuse injuries.

An emerging trend, quantifying sport actions with body-worn inertial measurements units (IMU), enables the assessment of athletes in ecologic conditions [17,18]. The metrological issues related to the use of wearable sensors for sport performance assessments have been the focal point of different research works: even though magneto-inertial technology allows monitoring the performance of athletes of all levels, especially when complemented with a sensor fusion network, there is a need for further research on the ease of use and error compensation to provide coaches and practitioners with informative and concise metrics [19,20,21,22]. The use of inertial units also raises technical issues to extract meaningful data from a broad class of signals (acceleration, angular velocity, magnetic field orientation, etc.) which are often prone to noise, non-linearities, and measurement inaccuracies. These characteristics might practically limit the usability of results in specific conditions. In the case of changes of direction, obtaining running speed analytically from one inertial unit and then applying linear equations to estimate the related energy cost in [8] appears practically unfeasible due to inherent biases and drifts.

A way to overcome these limitations is to apply machine learning techniques to IMU data. Supervised machine learning algorithms take a known set of input data (called predictors) and know responses and train a model to generate predictions from new data [23]. These techniques have been applied in team sports to quantify movement patterns during training and competition, like physical output and tackling impacts in rugby and Australian football [18,24], player load [25] or deceleration before turns in soccer [26]. However, the estimation of the energetics associated with 180-degree cutting actions in team sports has not been investigated. This study intends to introduce the application of machine learning models to detect direction, speed and external mechanical work associated with 180-degree CoDs, by using only data coming from a single inertial unit. We hypothesize that a unique sensor placed close to the core (pelvis) could capture the key information on athletes’ actions during these tasks. Our complementary aim is to show that the combination of regression analysis technique and easily available sensors can provide coaches and practitioners with a wealth of information about such crucial game actions.

## 2. Materials and Methods

### 2.1. Experimental Procedures and Equipment

All tests were performed in the morning within two weeks after the end of the regular season. The experimental setting was a full motion capture laboratory equipped with an eight-camera system (sampling frequency: 100 Hz; Smart-Dx, BTS Bioengineering, Milano, Italy). A set of 14 reflective markers (diameter: 15 mm) were positioned on the skin in the following anatomical landmarks: tragi, acromia, olecranons; radius styloid processes; greater trochanters; femoral lateral epicondyles; and lateral malleoli (additional markers were added for further biomechanical investigations, but they were not considered in the current study). A six-axes IMU (GaitUp Physilog 5, Lausanne, Switzerland) was fixed to the shorts with a plastic clip close to the sacrum marker. Inertial sensor settings were: sampling frequency 512 Hz, measurement range ±2000 degrees/s (gyroscope) and ±16 g (accelerometer). The *x*-axis of the sensor reference frame pointed backwards, the *y*-axis upwards and the *z*-axis to the subjects’ left. The unit also included a barometer with a sampling frequency of 64 Hz.

**Figure 1:**
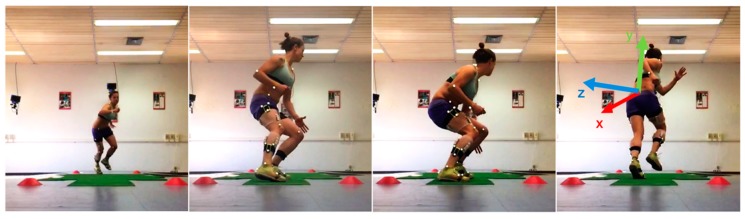
Experimental setting and turn (180-degree change of direction) action. In the right picture, the orientation of the sensor reference frame is displayed.

Ambient temperature was 22–24 °C. Participants wore minimal sports clothing and running shoes. They were first acquainted with the experimental procedures; after a 10 min warm up supervised by a professional strength and conditioning coach, participants completed a 5 m shuttle-run test (Figure 1) at the average speed of 70% of their maximal aerobic speed, as detailed in [7]. Maximal aerobic speed is the lowest running speed at which the maximum oxygen uptake occurs, and it was estimated with aerobic power tests (Yo-Yo intermittent recovery test [27]) throughout the season. Average running speed was 2.5 ± 0.2 m/s. Athletes had to keep running to exhaustion, i.e., when they could not reach the end lane by the acoustic signal—pacing the shuttle rhythm—for two consecutive times.

### 2.2. Study Design and Participants

This observational case-series study involved 13 female soccer players (age 23.6 ± 3.3 years, body mass: 59.0 ± 7.3 kg, height: 1.66 ± 0.05 m, body mass index: 21.2 ± 1.9 kg/m^2^), playing for elite clubs competing in the first and second Italian league. All participants were judged by a medical doctor not to have any restriction to sports practice, had no injuries in the 12 months preceding the test, and signed a written informed consent after a detailed explanation of the aims, benefits and risks of this study. The study was approved by the Institutional Ethics Committee (n. 1/2016) and was conducted according to the Declaration of Helsinki.

### 2.3. Data Processing and Features Engineering

**Figure 2:**
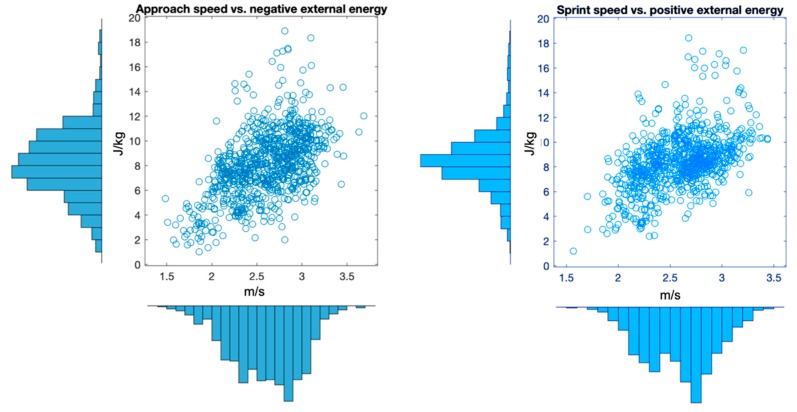
Negative (**left**) and positive (**right**) external mechanical energy computed in the two second window across the turn as a function of running speed before (**left**) and after (**right**) the turn. The distribution of the variables is also displayed on the edges.

Custom routines were developed within Matlab (v. 2018b, The Mathworks Inc., Natick, MA, USA). The three-dimensional coordinates of body center of mass (CoM) were obtained using the segmental centroid method, specifically validated for sports applications [28,29,30], after applying a low-pass, zero-lag second order Butterworth filter (cut-off frequency: 15 Hz) to the raw marker trajectories. CoD events were easily identified with the peaks of the CoM position in the running direction (*CoM_x_*). Mass-specific CoM external energy was computed according to classical physiology texts [31]:

(1) $$E_{ext}=\frac{1}{2}v_{CoM}^{2}+gh_{CoM}$$

where ***v****_CoM_* is the norm of instantaneous CoM speed, and *h_CoM_* is its vertical height. Positive (negative) mechanical work (*W^+/−^*) was then obtained as the sum of positive (negative) changes of *E_ext_* [31] in the two second window across the turn. Also, for each turn, we computed CoM approach speed (1 s before the turn, *v_before_*), and CoM speed during acceleration (1 s after the turn, *v_after_*). These four variables, alongside the side of the pivoting limb (right or left), constitute the set of known targets (responses). Their distribution is illustrated in Figure 2: as negative external work is required to decelerate the CoM before the turn, and positive work is needed to accelerate it back in the new direction, *W^−^* was represented relative to *v_before_*, and *W^+^* to *v_after_*.

**Figure 3:**
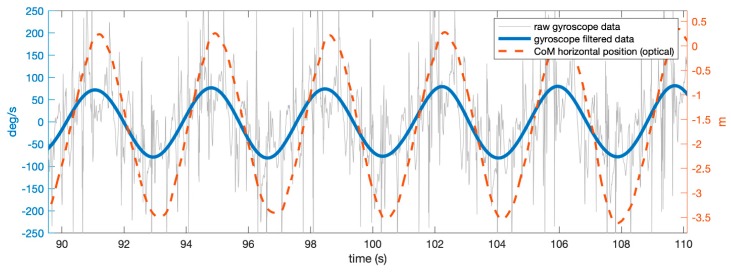
Events detection, based on center of mass (CoM) horizontal position (red curve, referred to the origin of the laboratory global reference system) and raw/filtered gyroscope rotation around the vertical axis (gray and blue, respectively). The autocorrelation function between the two allowed us to synchronize the two measurement systems.

Eighteen features (predictors) were extracted from IMU data. We first had to detect the same CoD events in the IMU readings: smoothed (Butterworth, zero-lag low-pass filter, f_cut_ = 0.5 Hz) angular velocity around the *y* (vertical) axis was particularly suitable for this, as it showed clear peaks in correspondence to turns (Figure 3). Matching between events timing was obtained by computing the cross-correlation between the resampled and rectified gyroscope and CoM trajectory and shifting them in time by an offset equal to the lag corresponding to maximum cross-correlation (Figure 3). The average CoD events detection error was 0.001 ± 0.102 s. Likewise for optical data, a two second window across this event was considered for the following computations. Static biases on each channel of the inertial sensors were obtained with a 30 min test with the unit kept still. We then subtracted these values from the accelerometer and gyroscope readings, which were additionally filtered with a fourth order Butterwort filter (cut-off frequency: 128 Hz).

The first feature F_1_ was the sum of the root mean square of changes in acceleration and deceleration per second, also known as ’player load’, a metric commonly used to calculate the load or activity level of athletes in team sports [25]:

(2) $$player\;load=\overset{1023}{\underset{t=1}{\sum }}\sqrt{{\left(a_{x,t+1}-a_{x,t}\right)}^{2}+{\left(a_{y,t+1}-a_{y,t}\right)}^{2}+{\left(a_{z,t+1}-a_{z,t}\right)}^{2}}$$

Other accelerometer features were the trapezoidal numerical integral of each axes positive and negative acceleration (F_2−7_). Features F_8−10_ were the integral of each gyroscope axis. Features F_11−13_ and F_14−16_ were the root mean square, the skewness and the kurtosis of the norm of the accelerometer and gyroscope readings during the two second turn window. Skewness describes the symmetry of the acceleration and angular velocity signal distributions and is given by:

(3) $$skewness=\frac{E{\left(x-\bar{x}\right)}^{3}}{\sigma^{3}}$$

The kurtosis quantifies the extent to which the acceleration and angular velocity signals are peaked or flat with respect to a normal distribution:

(4) $$kurtosis=\frac{E{\left(x-\bar{x}\right)}^{4}}{\sigma^{4}}$$

In Equations (3) and (4), *E* is the expected value, $\bar{x}$ is the mean and σ is the standard deviation of the signal in the two second window [32]. These two features quantify the degree of distortion with respect to a normal distribution of acceleration or angular velocity data series: for instance, an abrupt braking action would contain more negative than positive acceleration values, and so it would be highly skewed.

The last two features F_17_ and F_18_ were obtained from the filtered barometer output (low-pass tenth order Butterworth filter, cut-off frequency: 1 Hz) and were the difference between the mean sea level altitude (computed from ambient pressure) at the CoD event and one second before or after the CoD, respectively. Table 1 provides an overview of features and responses. Before further processing, outliers were removed when examples were outside the variable’s mean ± 3 standard deviations, and the coefficient of variation (CV) was computed for each variable.

### 2.4. Regression and Classification Models

For the prediction of W^+^, W^−^, *v_before_* and *v_after_*, we applied four supervised machine learning regression techniques:

1. Multiple linear regression, modeling the linear relationship between predictors and the response (dependent) variables.
2. Support vector regression (SVR): this technique is based on support vector machines (SVM), which in turn construct hyperplanes to define decision boundaries in a multi-dimensional space. SVR computes the parameter of a function *f(x)*, where ***x*** is the matrix of predictors, fitting the input data with the most ε-deviation from the target *y* (response). As SVR is particularly suited to handle non-linear tasks, in this study we chose a Gaussian kernel.
3. Boosted trees (BT): classification or regression models are in the form of a tree structure, which is built top-down from a root node, and involves partitioning data into subsets that contain common features based on the level of information gain, i.e., a decrease in entropy after a dataset is separated [33]. Boosted trees are an extension of decision trees that aggregate an ensemble of decision trees into a unique result, which reduces the chance of overfitting. The number of learners (trees) set in this study was 40.
4. Artificial neural networks (ANN): a feedforward network consisting of an input, a hidden and an output layer was designed. Neurons (*n* = 40) in the hidden layer process the input features in accordance to hyperbolic tangent sigmoid functions. The output layer is a single neuron which returns the estimated (predicted) response. The back-propagation learning algorithm was used to update the weights and biases of the ANN. Input data was split into three subsets: 70% for training, 15% for testing and 15% for validation.

Classification models to determine turn direction (right or left) matched the previous techniques and included: (1) linear discriminant analysis, (2) SVM, (3) BT, (4) ANN.

### 2.5. Validation

To evaluate the predictive accuracy, models 1–3 underwent a standard 10-fold cross-validation procedure: data were randomly partitioned into two sets, the first was used for training, while the second was used for validation. This process was repeated 10 times, by randomly selecting the training and validation portions [34]. The means of the 10 classification accuracy rates were taken as an unbiased estimate of the model for the complete dataset. Root mean square error (RMSE), mean absolute error and coefficient of determination (R^2^) were computed as performance metrics for regression models. Classification models were evaluated in terms of accuracy, sensitivity, specificity and area under the receiver operating characteristic curve (AUC).

## 3. Results

Th shuttle run test duration was on average 158.4 ± 65.1 s, with 72 ± 30 turns per participant. Overall, we collected 937 cutting maneuvers. Outlier removal led us to exclude 32 of them. Feature means, standard deviations and ranges are shown in Table 1: while the variability of speed was below 15%, that of positive/negative mechanical work reached 33%; the highest variability in predictors was contained by gyroscope integrals and RMS, the lowest in the acceleration integrals.

Turn direction was detected with good accuracy (98.4%, Table 2) by linear discriminant analysis (sensitivity = 97.3%, specificity = 99.6%, AUC = 1.00), while with the other methods (boosted trees, SVM and ANN), we obtained perfect classification (accuracy = 100%, AUC = 1.00).

**Figure 4:**
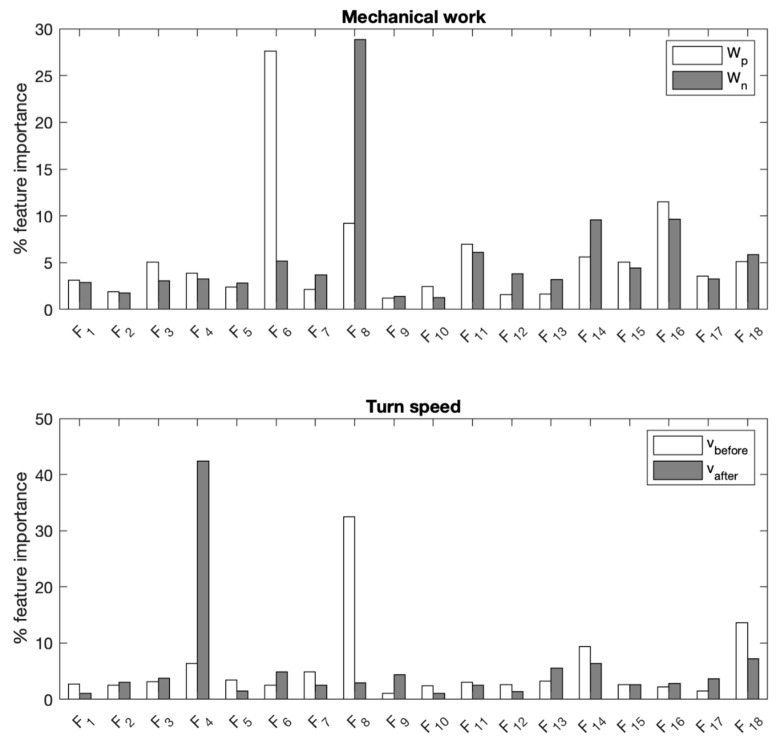
Feature importance returned by boosted trees models when predicting positive/negative mechanical work (W_p_ and W_n_, top), and approach/sprint running speed (v_before_ and v_after_, bottom). Refer to Table 1 for the description of features *F_i_*.

The best performance in predicting the mechanical work in decelerations and accelerations was achieved by SVR models, with a moderate R^2^ and an error of about 15% (Table 2). ANNs best predicted incoming and sprint speed with a moderate to substantial R^2^, and an error lower than 10% (0.15–0.18 m/s). Feature importance for BT is reported in Figure 4.

## 4. Discussion

The combination of accelerometer-, gyroscope- and barometer-based features and relatively simple machine learning models enabled us to estimate key kinematic and energetic characteristics of 180-degree turns with an error of about 15%. Features were obtained from a single pelvis-mounted unit, and no prior calibration procedure was required. In addition, we limited signal processing to a few basic steps, with only standard low-pass filtering being applied to sensor readings: this potentially increases the generalizability to different units and vendors.

### 4.1. Turn Direction

As CoDs and sidestepping maneuvers impose a high muscular and mechanical load on lower limb structures, knowing the intensity and direction of these actions might help in preventing unilateral overloading and in turn potential injuries [2,35]. Turn side (direction) was satisfactorily estimated even with linear discriminant analysis, which makes the adoption of BT, SVM and ANNs redundant for this specific task.

In fact, the combination of gyroscope features F_11_ (integral of the angular velocity around the pelvic anteroposterior axis) and most importantly F_12_ (integral around the pelvic mediolateral axis) returned almost perfect classifications. In other words, pelvis rotation captures the most relevant information about turning direction and could be easily implemented in existing global positioning system (GPS) activity trackers [36]. This sensor location allows for the implementation of smart apparel, that unlike smartwatches or wristbands, can be worn during games. A potential improvement to this model could be the classification among multiple classes of directional changes, i.e., 45–90 degree, 90–120 degree and 120–180 degree CoDs. Further, it is likely that the classification performance would decrease when positioning the sensor on other less favorable—limited to turn direction classification—segments such as feet or shanks.

### 4.2. Turn Speed and Mechanical Energy

Turning speed is related to the energy dissipated during the deceleration (braking) phase, and thus impacts on the risk of ligamentous lesions, especially at the knee level [37]. Once a directional change was detected, the proposed models were successfully able to estimate turn speed with an error below 0.2 m/s, which is comparable to 10 Hz GPS error in common sports applications [38,39]. However, rapid directional changes usually degrade GPS accuracy [38]: obtaining speed during CoDs still represents a challenge for such systems [40], whose weaknesses can be mitigated by adding or integrating data from inertial sensors [36,41].

The best prediction performance of ANNs showed that a linear relationship between predictors and response (i.e., multiple linear regression) was outperformed by non-linear models: ANNs can detect complex nonlinear interactions between inputs and targets [42]. However, the lack of transparency in the mathematical models of ANNs hinders the interpretation of their output. Decision trees showed slightly lower performance, but the model structure of the entire procedure can be followed and interpreted: in BT models, prediction appeared highly dependent on positive mediolateral acceleration (F_4_) for sprinting speed, and acceleration skewness for approach speed (Figure 4).

A similar reasoning applies to *W_p_* and *W_n_*, which were better predicted by SVR with Gaussian kernel. Mechanical energy during a CoD is an indirect measure of its associated metabolic cost. Computed positive/negative external CoM work was in line with previous investigations from different groups [8,16], which ensures the predictions were constructed on a solid basis. The prediction error (1.1–1.4 J) can be considered acceptable as it allows us to get a realistic measure of the amount of energy involved in the braking/propulsive actions, which is highly dependent on running speed.

### 4.3. Limitations and Perspectives

Turning event detection was particularly easy in the featured experimental protocol, which involved repeated 180-degree turns with just few running steps in between: further developments should include various angles of approach, and potentially a less homogeneous sample to increase the model’s generalizability. Compared to the reference optical system, CoD events were determined with a variability of about 0.1 s, which is of the same order of magnitude of the stance phase of the pivoting limb (~0.3 s [14]). However, we chose to compute features over a two second span; thus, any overlaps should be in the range of 5% of the whole window. Although data were processed off-line, once a turn is detected it would be relatively straightforward to apply the regression algorithms to moving windows containing updated data streams.

More complex features could be also added to exploit the IMU three-dimensional orientation (i.e., quaternions): however, (i) accurate long-term orientation tracking based on a unique inertial sensor is not trivial and (ii) we intentionally limited feature processing to variables that could be easily computed on a portable device. In addition, the proposed models did not rely on anthropometric information (height, weight, BMI, etc.), that were purposefully excluded from predictions.

Even if a more general scope should be adopted for practical on-the-field implementations, this paper showed that the extraction of meaningful information on turn kinematics and energetics is highly viable with a data-driven approach using commercially-available units and established regression and classification techniques. The current study represents a further step towards the accurate, ecological quantification of the key features of changes of direction in team sports.

## Figures and Tables

**Table 1:** Overview of the five responses (*R_i_*, where R_5_ is a categorical variable) and of the 18 features (*F_i_*) used to train the models.

Variable	Description	Unit	Mean	SD	CV	Min	Max
R_1_	Positive work	J/kg	8.49	2.35	0.28	1.18	18.44
R_2_	Negative work	J/kg	8.17	2.73	0.33	1.02	18.91
R_3_	Speed before turn	m/s	2.60	0.38	0.15	1.49	3.69
R_4_	Speed after turn	m/s	2.59	0.33	0.13	1.57	3.44
R_5_	Turn side	cat.	-	-		-	
F_1_	Player load	A.U.	190.6	62.6	0.33	67.3	407.4
F_2_	Positive acc_x_ integral	m/s	325.8	86.0	0.26	125.7	584.0
F_3_	Positive acc_y_ integral	m/s	528.00	96.1	0.18	267.4	846.3
F_4_	Positive acc_z_ integral	m/s	238.7	67.6	0.28	82.0	435.1
F_5_	Negative acc_x_ integral	m/s	−328.7	89.4	0.27	−587.1	−107.8
F_6_	Negative acc_y_ integral	m/s	−508.8	89.2	0.18	−810.1	−263.4
F_6_	Negative acc_z_ integral	m/s	−251.7	79.0	0.31	−504.5	−93.4
F_7_	Norm acc RMS	m/s^2^	7.59	3.57	0.47	1.19	21.04
F_8_	Acceleration skewness	-	4.95	1.35	0.27	2.08	9.00
F_9_	Acceleration kurtosis	-	35.8	18.5	0.52	7.6	98.4
F_10_	Gyroscope_x_ integral	rad	9.9·10^3^	1.4·10^4^	1.44	−2.9·10^4^	4.7·10^4^
F_11_	Gyroscope_y_ integral	rad	0.9·10^3^	6.7·10^4^	n.a.	−8.5·10^4^	8.6·10^4^
F_12_	Gyroscope_z_ integral	rad	−45.3	3.8·10^4^	n.a.	−6.0·10^4^	6.1·10^4^
F_13_	Gyroscope norm RMS	rad/s	2.4·10^3^	1.7·10^3^	0.69	0.3·10^3^	7.9·10^3^
F_14_	Gyroscope skewness	-	4.64	1.61	0.35	0.41	9.31
F_15_	Gyroscope kurtosis	-	32.8	20.1	0.61	2.41	104.0
F_17_	$\Delta_{baro}$, before	m	−0.39	0.26	0.65	−1.08	0.36
F_18_	$\Delta_{baro}$, after	m	0.41	0.26	0.62	−0.35	1.12

$\Delta_{baro}$: altitude difference between before/after 1 s. acc: acceleration; A.U. arbitrary units; cat.: categorical variable. CV: coefficient of variation; RMS: root mean square; SD: standard deviation; n.a.: not applicable (bimodal variable, CV > 50).

**Table 2:** Performance of regression models. In bold, the best model for each response, in terms of root mean square error (RMSE).

Response	Regression Model	R^2^	RMSE	MAE
Positive work (J)	Multilinear regression	0.36	1.96	1.30
	**Support vector regression**	**0.43**	**1.85**	**1.14**
	Boosted trees	0.39	1.91	1.22
	Artificial neural network	0.39	2.40	1.80
Negative work (J)	Multilinear regression	0.35	2.27	1.66
	**Support vector regression**	**0.42**	**2.14**	**1.41**
	Boosted trees	0.41	2.16	1.49
	Artificial neural network	0.44	2.34	1.84
Speed before (m/s)	Multilinear regression	0.53	0.26	0.21
	Support vector regression	0.65	0.23	0.17
	Boosted trees	0.60	0.24	0.19
	**Artificial neural network**	**0.66**	**0.23**	**0.18**
Speed after (m/s)	Multilinear regression	0.47	0.25	0.20
	Support vector regression	0.65	0.20	0.15
	Boosted trees	0.62	0.21	0.16
	**Artificial neural network**	**0.69**	**0.19**	**0.15**

MAE: mean absolute error; R^2^: coefficient of determination.
